# Thermal oscillations in rat kidneys: an infrared imaging study

**DOI:** 10.1098/rsta.2008.0117

**Published:** 2008-07-23

**Authors:** Alexander M. Gorbach, Hengliang Wang, Eric Elster

**Affiliations:** 1 National Institute of Biomedical Imaging and Bioengineering, National Institutes of HealthBuilding 13, Room 3N-11, Bethesda, MD 20892–5766, USA; 2 Naval Medical Research CenterSilver Spring, MD 20910, USA

**Keywords:** infrared imaging, blood flow, thermal oscillations, autoregulation, synchrony

## Abstract

A high-resolution infrared (IR) camera was used to assess rhythmicity in localized renal blood flow, including the extent of regions containing nephrons with spontaneous oscillations in their individual blood flow. The IR imaging was able to follow changes in rat renal perfusion during baseline conditions, during occlusion of the main renal artery and during the administration of either saline or papaverine. Concurrent recordings were made of tubular pressure in superficial nephrons. Spontaneous vascular oscillations centred around 0.02–0.05 Hz and approximately 0.01 Hz could be detected reproducibly by IR imaging. Their spectral characteristics and their response to papaverine were in line with tubular pressure measurements. The intensity of and synchrony between thermal signals from different local areas of the kidney may allow, after surgical exposure, non-invasive imaging of functional clusters involved in renal cortical blood flow. Through visualization of the spatial extent of thermal oscillations, IR imaging holds promise in assessing kidney autoregulatory mechanisms.

## 1. Introduction

Single nephrons in mammalian kidneys have been observed to regulate their own blood flow via two mechanisms: (i) tubuloglomerular feedback (TGF), which senses flow rate-dependent changes in the concentrations of certain electrolytes in the tubular fluid, and (ii) a myogenic mechanism, which senses vascular hydrostatic pressure in the afferent arterioles (Just 2007; Marsh *et al*. 2007). Nephrons can communicate with each other by means of vascular signalling initiated by the TGF mechanism and propagated electrotonically and decrementally along the vascular wall (Kallskog & Marsh 1990). Both TGF regulation and the myogenic mechanism are oscillating systems that interact with each other within single nephrons (Kallskog & Marsh 1990) and, by virtue of the vascularly propagated signals, between neighbouring nephrons (Sosnovtseva *et al*. 2004). Assuming that all or most nephrons oscillate and interact, synchronization is to be expected among nephrons in different cortical areas. Owing to limitations in the technique, synchrony and propagation phenomena have traditionally been studied experimentally only among small numbers of cortical nephrons.

We performed experiments using infrared (IR) imaging to assess blood flow on the entire exposed surface of kidneys and to establish the pattern of nephron–nephron interaction in the superficial part of a kidney cortex. Specifically, we attempted to find out whether all nephrons oscillate with only a single common frequency, such as the TGF-related frequency of approximately 0.02–0.05 Hz, and, if so, whether phase synchronization occurs.

## 2. Material and methods

An IR camera (FLIR, California, USA) with enhanced optics (2× IR Microscope, Diop, USA) and ENVI image analysis software (ITT Visual Information Systems, Boulder, CO, USA) was adapted to assess rat renal perfusion during (i) the baseline condition, (ii) the administration of saline and papaverine through a femoral artery, (iii) occlusion of the main renal artery with a vascular clip, and (iv) reperfusion, including post-occlusion reactive hyperaemia (PORH). The camera (14 bits, 0.02°C thermal resolution, 320×256 pixels per image, 1.0–2.0 Hz acquisition rate), which was sensitive to the passive emission of IR photons with 3–5 μm wavelength, was positioned 7 cm above the exposed left kidney, while the kidney's movement was mechanically restricted by a plastic holder. During imaging sessions, the animals (*n*=8) were under general anaesthesia (sevoflurane) and were placed on a heater-equipped support table. Total renal blood flow was recorded by a transit-time ultrasound probe placed around the left renal artery (Transonic Systems, USA). The common carotid artery and the jugular vein were cannulated for measurement of arterial blood pressure and infusions, respectively. Proximal tubular pressure in a superficial nephron was recorded with a micropipette connected to a servo-nulling device (Baumbach Electronics, Denmark) and sampled at 1.0 kHz (Sorensen *et al*. 2000). To minimize the impact of air convection on IR measurements, the surgical table and the micropipette with a manipulator were placed into a foam box with a hole for the IR microscope lens.

A three-step approach was designed for the analysis of collected imaging datasets.

- *IR images* were aligned for each imaging session to remove motion artefacts. First, a video frame was chosen to serve as a reference for all other images in the same session. Next, each imaging session was divided into trials. Each trial consisted of 250 or 500 images acquired with 1.0 and 2.0 Hz acquisition rates, respectively. Next, at least three fiducial points were selected for each kidney (such as points of maximum curvature of blood vessels or kidney edges, or blood spots) in each image of a trial. An image registration algorithm was designed to find the maximum cross-correlation coefficient between the fiducial points of the reference frame and those of each other frame. This enabled the calculation of the translational shifts required to register the points in each frame with the reference frame. The ENVI imaging registration tool was then able to calculate the affine parameters needed to align all frames in a trial using a non-rigid body registration approach. To validate the image registration quality, a performance algorithm was developed. A kidney mask was extracted from the reference frame using the ENVI software. The maximum cross-correlation coefficient between every adjacent pair of frames within the mask border was calculated again, and frames with a large shift (three or more pixels) were selected from the image trial for further alignment. The image alignment procedure was repeated again for the selected frames, but with a different set of fiducial points, until the performance algorithm showed a well-aligned (3–5 pixel shifts per frame) image trial. The final image sequence was recreated by sequentially concatenating the aligned trials.
- *Temperature profiles* (IR intensity versus time) were obtained for the following regions of interest (ROIs): (i) the entire kidney ROI and (ii) a local ROI (3×3 pixels) within each IR frame of an imaging sequence. The profiles allowed us to evaluate temporal aspects of thermal changes, such as the mean temperature changes over time, and localized oscillations of temperature for each ROI.
- *Spectral analysis* was applied to identify the oscillation frequency range for the temperature profiles extracted from the entire kidney ROI, for each imaging sequence. A first-order polynomial detrending algorithm (ENVI/IDL software) was applied to remove trends that might be present across successive trials. Next, a power spectrum was calculated (Stoica & Moses 1997) by applying a fast Fourier transformation (FFT) to the 250 or 500 points of the data in each thermal profile of each trial. After the calculation of the mean power spectrum for the entire kidney, the range of frequency oscillations was identified.

Time series extracted from the temperature profiles with a 3×3 smoothing window and single micropipette tubular pressure records were compared using the FFT and continuous Morlet wavelet transform (CMWT) methods. The CMWT method allowed the calculation of (i) continuous wavelet spectra, (ii) a time-averaged wavelet phase synchronization (TWPS) index, and (iii) time-averaged wavelet phase coherence (TWPC; Bandrivskyy *et al*. 2004).

The CMWT of a signal *x*(*t*) was defined by

$$X(\omega ,t)=\int_{-\infty }^{\infty }\Psi_{\omega ,t}(u)x(u)\text{d}u,$$

where *Ψ* represents the Morlet window function.

For each time *t*_*n*_ and frequency $\omega_{k}$, the complex value of the wavelet transform $X(\omega_{k},t_{n})$ was obtained as $X(\omega_{k},t_{n})=a_{k,n}+\text{i}b_{k,n}$. The wavelet spectral magnitude may then be calculated as

$$SP_{k,n}=|X(\omega_{k},t_{n}){|}^{2}=a_{k,n}^{2}+b_{k,n}^{2}$$

and the phase of the particular frequency was determined by

$$\phi_{k,n}=\text{arctan}(b_{k,n}/a_{k,n}).$$

For two signals *x*_1_(*t*) and *x*_2_(*t*), the relative phase difference given by $\Delta \phi_{k,n}=\phi_{2k,n}-\phi_{1k,n}$ can be computed and the TWPS index was then defined as

$$S_{\phi }(\omega_{k})=\bar{\text{cos}(\Delta \phi_{k,n})},$$

where $\bar{\text{cos}(\Delta \phi_{k,n})}$ is the mean of $\text{cos}(\Delta \phi_{k,n})$. The higher the TWPS index, the closer the phase synchrony is between the two signals.

The TWPC was calculated as

$$C_{\phi }(\omega_{k})=\sqrt{{\bar{\text{cos}(\Delta \phi_{k,n})}}^{2}+{\bar{\text{sin}(\Delta \phi_{k,n})}}^{2}},$$

where $\bar{\text{sin}(\Delta \phi_{k.n})}$ is the mean of $\text{sin}(\Delta \phi_{k.n})$.

Whereas the spectral ranges for temperature and tubular pressure time series were estimated with FFT-based power spectral analysis, the use of continuous wavelet spectra allowed the assessment of the duration for these oscillations.

To find out whether nephron–nephron synchronization occurred, the TWPS index was calculated between the temperature profiles extracted from the centre of each of the warmest areas of an IR image and all other temperature profiles. Next, the TWPS indices were presented as synchronization maps that varied with frequency. Finally, the TWPC indices were calculated to quantify the relationships between IR temperature and tubular pressure signals.

From the beginning of the investigation, our attention was focused on the potential impact of 1/*f* thermal noise on the spectral results. In particular, calibration experiments and calculations were conducted to minimize the potential impact of noise and spectral errors related to the IR radiative effects from the wet kidney surface. In addition to a 20 min standard IR calibration procedure with a black-body calibration source, another procedure was implemented using a room-temperature water bath in a laboratory environment. Both procedures involved collecting a dataset of IR images over 20 min. The water bath approach allowed us to account for noise due to evaporative cooling from the wet surface of the exposed kidney. Effects of this type occur during all experimental conditions with exposed organs. Separate spectral magnitudes were calculated for these datasets at different frequencies, and the spectral magnitudes from the water bath dataset were chosen as lower thresholds for wavelet spectral analysis.

During the experiments on animals, IR measurements were started immediately after oscillations were detected in proximal tubular pressure and were limited to 18 min per imaging session.

## 3. Results

**Figure 1:**
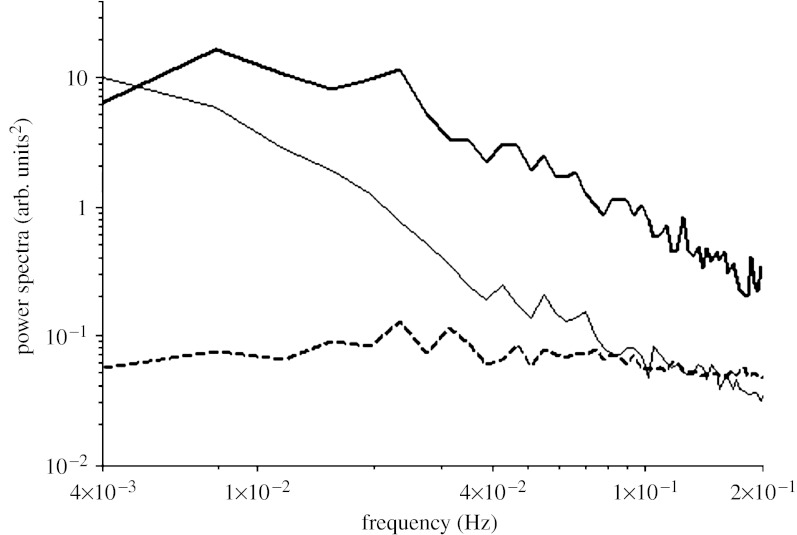
Averaged power spectra for the IR signal collected from the standard black-body calibration source (thick dashed line), water at room temperature (thin solid line) and the 3×3 pixel ROI (thick solid line) in the central area of rat kidneys (*n*=5).

White noise was measured in signals from a black-body calibration source (figure 1, thick dashed line). The 1/*f* characteristic noise was then measured in signals from the water bath (figure 1, thin solid line). Two distinct peaks close to 0.025 Hz and approximately 0.01 Hz, followed by 1/*f* noise, were found in the exposed kidney (figure 1, thick solid line).

**Figure 2:**
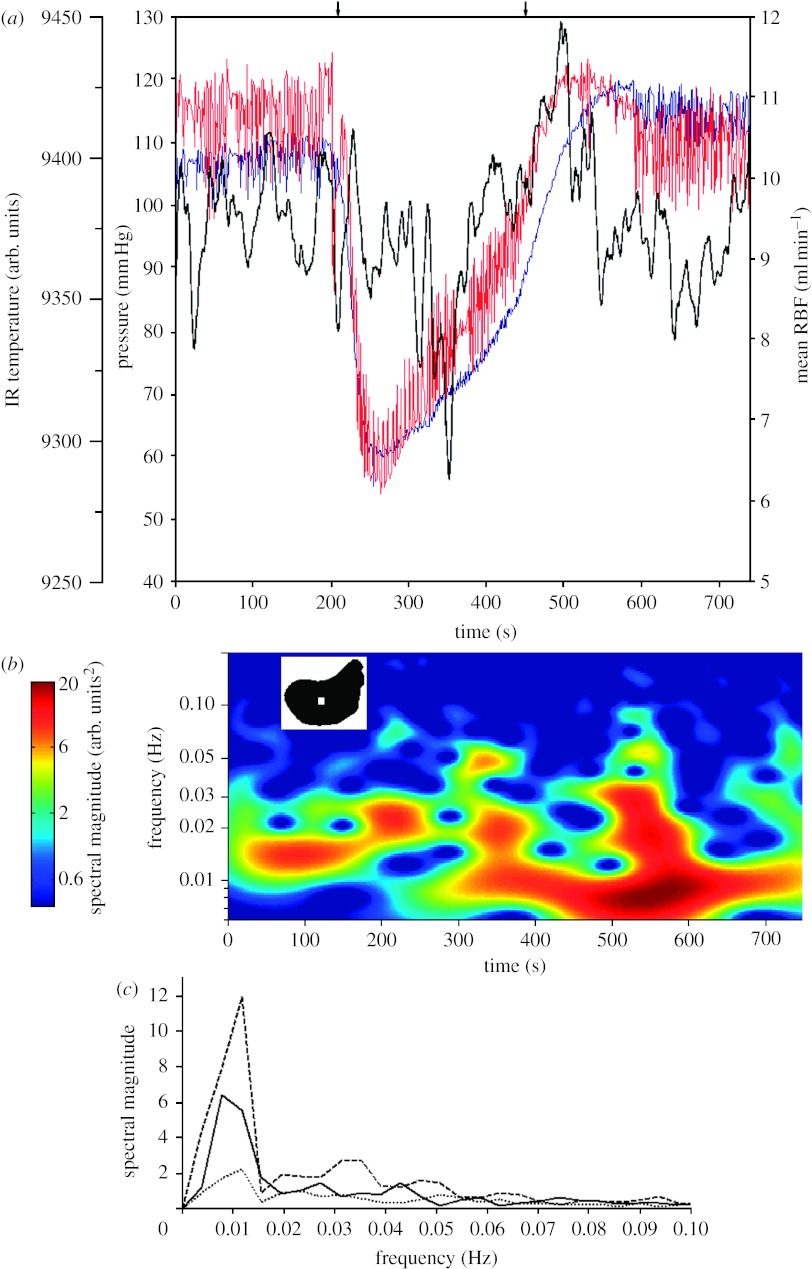
(*a*) Changes in arterial pressure (blue), renal blood flow (RBF; red) and IR temperature (black) during baseline, injection of papaverine and post-injection. A linear trend was subtracted from the raw IR signal. Injection start and stop times are marked by arrows at the top of the graph. (*b*) Spectral frequencies, their power and duration of oscillations calculated for a single IR temperature profile (ROI 3×3 pixels, shown in the kidney map inserted at the upper left) during the administration of papaverine. (*c*) IR signal mean power spectra (*n*=3) for baseline (solid line), papaverine injection (dashed line) and post-injection (dotted line) conditions.

The effect of the administration of papaverine (a vasodilator) on blood flow, pressure and temperature is shown in figure 2*a*. The injection of papaverine was initiated at 200 s and stopped at 450 s. A comparison of total renal blood flow (RBF) and arterial pressure with concurrent temperature changes during the administration of vasodilating substances showed a similarity in the deflection of all three signals between 200 and 500 s, as well as the presence of low-frequency oscillations in the IR signal. Figure 2*b* shows the dynamics of the magnitude and duration of these oscillations for each frequency for a single IR temperature profile during baseline, injection of papaverine and post-injection. Figure 2*c* shows the IR-averaged power spectra (*n*=3) for the same ROI during baseline, and during and after papaverine injection. The power spectral magnitude of very slow oscillations (VSOs), approximately 0.01 Hz, was significantly different for the following conditions: baseline, during injection, and after injection (*p*=0.015; one-way repeated ANOVA, Student–Newman–Keuls methodology). The power spectral magnitude of VSOs during baseline was significantly smaller than during drug injection (*p*=0.016). The power spectral magnitude of VSOs after papaverine injection was also significantly smaller than during papaverine injection (*p*=0.018). There was no significant difference for the power spectral magnitude of VSOs between baseline and post-injection (*p*=0.106), although the power spectral magnitude of VSOs during baseline was larger than that post-injection. There were no significant differences between the power spectral magnitudes of IR signals for the baseline, injection and post-injection conditions within the TGF frequency range (*p*=0.194).

**Figure 3:**
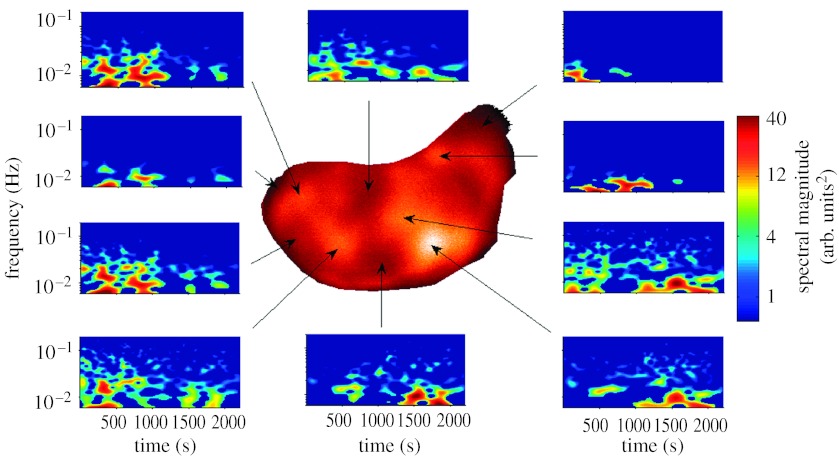
Frequencies, their spectral magnitudes and duration of the temperature oscillations in the kidney, acquired during baseline conditions (interval 0–2200 s). Plots were calculated for the IR-derived temperature profiles extracted from warm and cold areas (3×3 ROIs) on the kidney surface. The frequency and spectral magnitudes (the latter normalized to the scale of the FFT) are shown on logarithmic scales.

**Figure 4:**
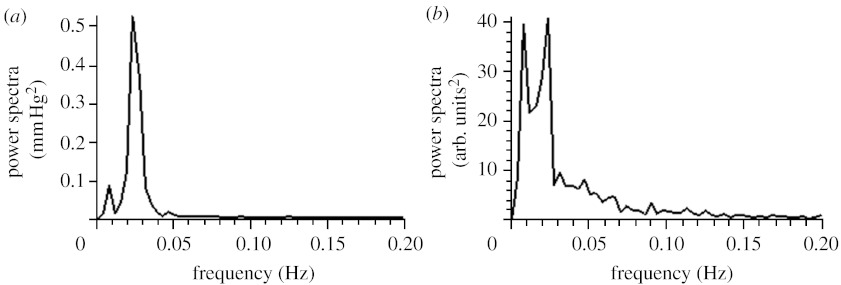
FFT-based power spectra of (*a*) tubular pressure and (*b*) IR-derived temperature profile, extracted from a 3×3 pixel ROI in close proximity to the tubular pressure micropipette (which was placed at the approximate centre of the kidney surface).

Figure 3 shows the spatial distribution of different frequency components and their dynamics during baseline (2200 s) IR image acquisition. The existence of two frequencies, a VSO frequency of approximately 0.01 Hz and a TGF frequency of approximately 0.02–0.05 Hz, for the blood flow oscillations was confirmed by FFT-based power spectrum analyses of tubular pressure and IR-derived signals (figure 4).

**Figure 5:**
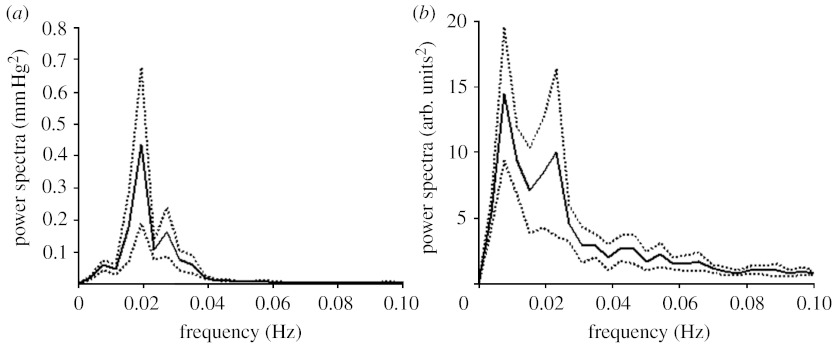
Averaged power spectra of (*a*) tubular pressure variations (*n*=5) and (*b*) the IR signal collected from ROIs (3×3 pixels) in the central region of the kidneys (*n*=5). Values are shown as mean±1 s.e.

High-intensity TGF oscillations of approximately 0.02–0.05 Hz and low-intensity VSOs of 0.01 Hz were found in proximal tubular pressure. The strongest IR-derived temperature oscillations were around 0.01 Hz (VSOs). Temperature oscillations in the TGF range of approximately 0.02–0.05 Hz were also present. Their power spectral components were the same as for the 0.01 Hz oscillations (figure 4*b*) or weaker (figure 5*b*).

Figure 5 shows the averaged power spectra of tubular pressure variations for five animals (figure 5*a*) and averaged power spectra of IR signals from the central region of kidneys in five animals (figure 5*b*, the same rats as the tubular pressure measurements). For the power spectra of tubular pressure, there are several peaks between 0.02 and 0.05 Hz. These result from variations in the TGF oscillation frequencies among the studied animals.

**Figure 6:**
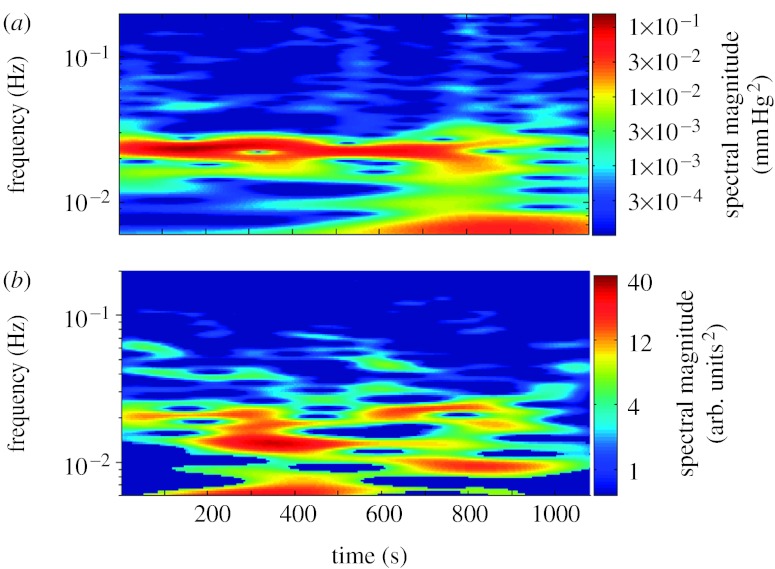
Spectral frequencies, their power and duration of oscillation calculated for (*a*) tubular pressure and (*b*) IR-derived temperature signals, acquired during the baseline interval (0–1100 s). The frequency and spectral magnitudes (the latter normalized to the scale of the FFT) are shown on logarithmic scales.

The frequency and power of oscillations changed with time, as can be seen by applying a wavelet transform to the tubular pressure and temperature signals for the baseline condition (figure 6), as well as to the temperature signals for different conditions (figure 8).

**Figure 7:**
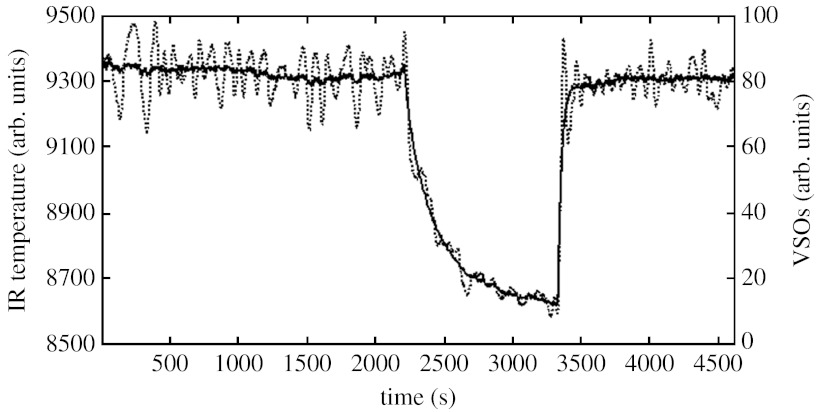
A representative IR temperature profile (solid curve) extracted from the same 3×3 pixel ROI in each of 4600 sequentially acquired (1/*s*) IR images. Temperature oscillations of the profile (dotted curve) are visualized by subtracting the trend, low-pass filtering (0.02 Hz cut-off frequency) and amplification (for clarity of demonstration).

A gross kidney perfusion deficit is visible in the IR images in real time, as well as measurable in the temperature profiles, after complete occlusion of the left renal artery. This is shown in figure 7, where the amplified oscillations are plotted on top of the IR temperature profile over a period of approximately 1¼ hours.

**Figure 8:**
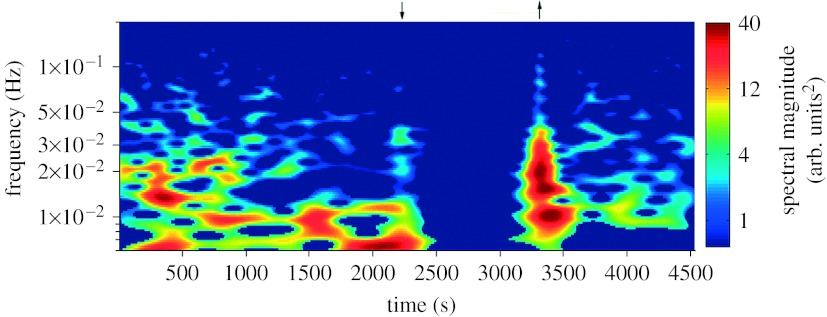
Spectral frequencies, their power and duration of oscillations calculated for an IR-derived single temperature profile (3×3 ROI). A total of 4600 IR images were acquired during the baseline kidney condition (0–2200 s), total renal occlusion (2200–3300 s), post-occlusion hyperaemia (3300–3500 s) and reperfusion (3500–4600 s). The onset of total renal occlusion and moment of reperfusion are marked by arrows at the top of the graph. The frequencies and spectral magnitudes (the latter normalized to the scale of the FFT) are shown on logarithmic scales.

As illustrated in figure 8, the oscillations ceased when the kidney was occluded, overshot during the PORH and partially returned to baseline during the reperfusion phase.

**Figure 9:**
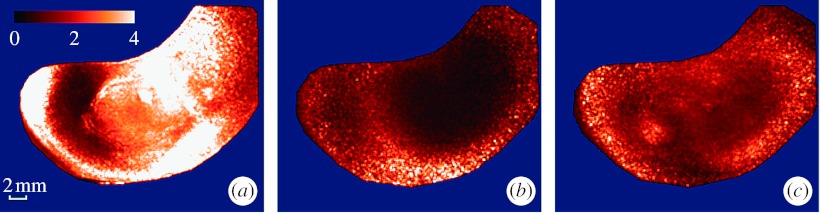
Spatial extent of the temperature oscillations (representative case). Values of each pixel in the images were calculated as the ratio between spectral powers at 0.01 Hz and approximately 0.02–0.05 Hz for (*a*) baseline, (*b*) occlusion and (*c*) reperfusion kidney conditions. A pseudo-coloured linear scale ((*a*) left upper corner) represents the ratio values.

In an attempt to characterize the spatial extent of the VSOs and TGF temperature oscillations for the baseline, occlusion and reperfusion conditions, the ratio between VSO power (spectra for 0.01 Hz) and TGF power (spectral range between 0.02 and 0.05 Hz) was calculated for each pixel of the kidney image. Figure 9 shows the results. The mean spectral ratio for the baseline condition (ROI at the central region of the kidney) is 2.55±0.49 (*n*=8, mean±s.e.). During total renal occlusion (*n*=3) with ischaemic times of 4, 18 and 22 min, the ratio becomes substantially smaller and its distribution shows a pattern with ‘granular’ spatial heterogeneity (figure 9*b*). More detailed analysis reveals that both the VSO and TGF intensities are decreased. However, the power of the VSOs was attenuated more than that of the TGF oscillations. The mean spectral ratio for the central area of the kidney during the post-reperfusion condition is 1.11±0.15 (*n*=3, mean±s.e.). The difference in the spectral ratio between the baseline and post-reperfusion conditions is significant (Mann–Whitney rank-sum test, *p*=0.008).

**Figure 10:**
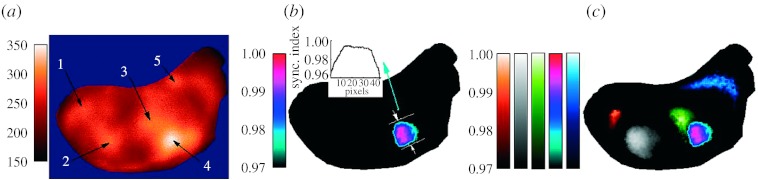
(*a*) Image with cold and warm areas (marked from 1 to 5) as calculated by subtraction of the IR image before reperfusion from the IR image during PORH. (*b*) TGF oscillation synchronization map and TWPS index changes (inset plot) between two arrows on the map, calculated for one of the warm areas (area 4 in (*a*)). (*c*) Localization of the highest synchrony for the TGF oscillations, calculated for the five warmest areas on the IR image (see (*a*)). Each cluster on the kidney image (r.h.s.) has an individual pseudo-coloured scale bar (l.h.s.).

Multiple temperature cold and warm areas were visible by IR on the kidney surface immediately after kidney reperfusion. Figure 10*a* shows the warm areas that were identified for further analysis. During the PORH, TGF oscillation synchronization maps showed that the highest local synchrony occurred around the centre of each of the five warm areas, with diminished values at the area edges (figure 10*b*). An integrated map (figure 10*c*) of synchronization, combining five independently calculated maps (such as the one in figure 10*b*), showed that the highest levels of TGF synchronization were confined to the immediate vicinities of the five warmest areas of the kidney.

**Figure 11:**
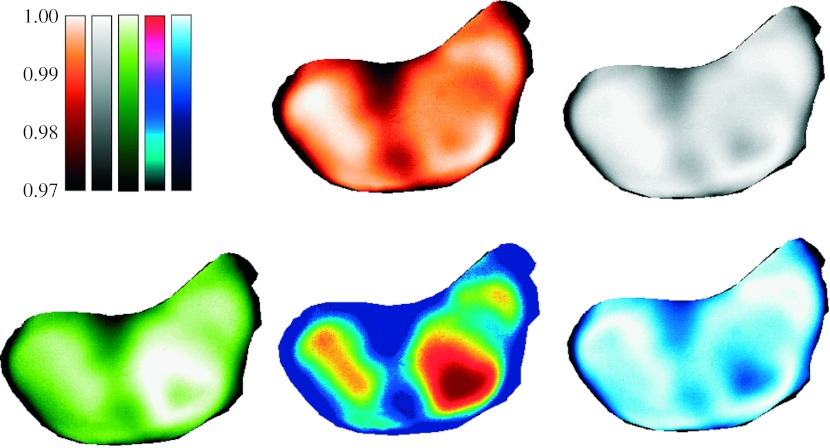
Localization of the highest synchrony for VSOs, calculated for the five warmest areas on the IR image (see figure 10*a*). Calculation of the TWPS index was performed with the same threshold as for figure 10*b*. Each kidney image has an individual pseudo-coloured scale bar (left). Note the extent of overlap in the synchronization maps.

The synchronization maps for the VSOs (figure 11) show a much greater and overlapping spatial synchrony than the synchronization maps for the TGF oscillations.

**Figure 12:**
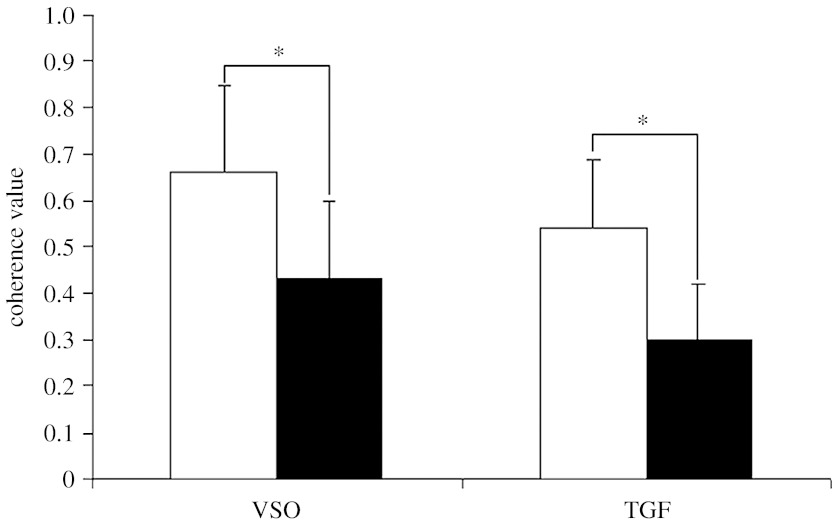
Coherence between tubular pressure and IR-derived oscillations (white bars), calculated for VSO and TGF frequencies (kidney baseline condition, *n*=15, Student's *t*-test, *p*<0.002). Black bars, surrogates.

For the baseline condition, the coherence between the IR and tubular pressure signals for the VSO and TGF oscillations are significantly higher than the coherence between their surrogates (figure 12). The surrogate data are generated using the iteratively refined amplitude-adjusted Fourier transform (IAAFT) surrogate data technique (Schreiber & Schmitz 2000). This method maintains an excellent approximation of both the power spectrum and the amplitude distribution of the original series.

## 4. Discussion

A high-resolution IR camera was used to assess microcirculatory fluctuations in localized renal blood flow and to learn more about the spatial extent of spontaneous oscillations in nephron blood flow. Arterial blood at core temperature is warmer than the exposed kidney surface, which becomes cooled by contact with room air and evaporation. Therefore, local microvascular blood flow can be used as an endogenous, natural thermal contrast agent for IR monitoring of the kidney during intraoperative conditions (Gorbach *et al*. 2003*a*,*b*).

Although the acquired unprocessed IR images did not allow for a clear detection of any specific pattern in the superficial blood flow, they did show changes in renal blood flow during total renal occlusion, as well as during administration of papaverine. Also observed were cold and warm patches after reperfusion, which are a projection of intralobular vessels on the kidney surface (Nordsletten *et al*. 2006).

Both the tubular pressure and IR measurements revealed, as expected, the presence of renal oscillations at a frequency of approximately 0.02–0.05 Hz. This is the frequency that was previously found in blood flow oscillations (Kallskog & Marsh 1990; Sorensen *et al*. 2000). Surprisingly, using the same methods, we also found a slower oscillation with a frequency of approximately 0.01 Hz.

Spectral analysis of the tubular pressure and IR signals revealed multiple, irregular bursts of oscillations (four to five bursts during two consecutive baselines) in the TGF and VSO frequency ranges. For the tubular pressure signals, the most powerful oscillations, with bursts, were observed at 0.008 and 0.022 Hz (figure 6*a*). For the IR signals, several other powerful frequency components were seen in addition to those frequencies (figure 6*b*).

Blood flow, particularly its VSOs, may arise in the larger intrarenal vessels. VSOs may be less easily detectable in the tubular pressure, owing to the relatively low amplitude. However, owing to their large spatial extent, VSOs become easily detectable on the renal surface with IR. By contrast, TGF oscillations may locally (at the level of the individual nephron) have quite large amplitude, which makes them easily detectable in the tubular pressure. However, owing to their local nature, TGF oscillations are more difficult to detect on the surface of the kidney with IR.

Although, in intraoperative conditions, the IR method is more specific to vascular-derived thermal changes (Gorbach *et al*. 2003*b*), we cannot exclude metabolic thermogenesis on the IR signal. The fact that VSOs are much more dominant in the IR measurements than in tubular pressure suggests that these oscillations may reflect an additional, metabolic source of heat and, therefore, lead to greater changes in the surface temperature. Heat is produced whenever energy is released within living tissue. It is inevitably generated by the chemical reactions that constitute cellular metabolism. Of the various pathways that are able to control the conversion of energy into either work or heat, at least two of them are well described in the literature as being involved in the process of non-shivering thermogenesis. These are the uncoupling proteins (UCPs) and the sarcoplasmic reticulum Ca^2+^-ATPase (SERCA). In both routes, the process of heat dissipation is initiated by the leakage of ions through the cellular membrane, protons in the case of the UCPs and Ca^2+^ in the case of the Ca^2+^-ATPase. The liberated heat was shown to be measurable from a single cell (Suzuki *et al*. 2007) and cell culture (Paulik *et al*. 1998; de Meis *et al*. 2005). UCPs and SERCA were found in longitudinal smooth muscle layers (Nibbelink *et al*. 2001; Wu *et al*. 2001) and, being involved in the regulation of thermogenic activity, may express an oscillatory behaviour (de Meis 2001; de Meis *et al*. 2005; Arruda *et al*. 2007).

In arterial smooth muscle, the elevation of intracellular Ca^2+^ is the immediate trigger for contraction, which ultimately determines vascular tone and rhythmic change in wall tension, i.e. vasomotion (Aalkaer & Nilsson 2005). Taken together, the above suggest that tight coupling between Ca^2+^, heat liberation and vasomotion allows us to hypothesize that a metabolic source of heat might be involved.

The temperature distribution visualized by the IR camera is determined primarily by the dynamic relationship between heat generation, i.e. heat contributed by local cellular metabolic activities, and heat dissipation, i.e. heat removed mostly by local blood flow (Gorbach 2004; de Meis *et al*. 2005; Suzuki *et al*. 2007). Probably, both heat components (metabolic and blood flow origin) are present in our IR measurements. However, the significant coherence between tubular pressure and IR-derived oscillations in our study (figure 12) suggests that the temperature oscillations are strongly coupled with blood flow. Endothelial-related vasomotion with the same slower frequency is a phenomenon seen in most, if not all, vascular beds, including the kidney, and might be the source of these oscillations (Bandrivskyy *et al*. 2004). In fact, Just (2007) convincingly demonstrated the presence of 0.01 Hz oscillations in total renal blood flow. All of the above could suggest that such a process takes place in vessels.

In this series of investigations, our principal observations were as follows. (i) IR-derived temperature changes are in line with arterial pressure and renal blood flow during total renal occlusion and the administration of papaverine. (ii) In addition to TGF oscillations, spontaneous VSOs can be consistently detected non-invasively in the rat kidney by IR imaging and tubular pressure. VSOs are more dominant in IR emission than in tubular pressure. (iii) The frequencies of VSOs are much below the cardiac (approx. 5.0 Hz) and respiratory (approx. 2.0 Hz) frequencies for the rat. (iv) TGF and VSO spectral frequencies, their power and the duration of the temperature oscillations (bursts) for different kidney areas change in time and are spatially heterogeneous. (v) The highest local synchrony in the IR-derived TGF oscillations is confined to the immediate vicinity of the warmest areas of the kidney cortex. The VSOs have a greater spatial synchrony than the TGF oscillations.

The highest local synchrony of the 0.02–0.05 Hz oscillations was confined to the immediate vicinity of the warmest areas of the kidney visible by IR after reperfusion. We hypothesize that active control of blood flow is exercised by the nephron clusters around a single radial artery, where the TGF frequency is approximately 0.02–0.05 Hz and endothelially related vasomotion is associated with the approximately 0.01 Hz oscillations. Oscillations around warm areas showed the highest synchrony with neighbouring regions and may represent functional clusters around radial arteries involved in TGF blood flow autoregulation. A study is in progress to understand the relationship between these demonstrated clusters of synchrony and phase propagation phenomena in the kidney cortex.

As previously reported in the literature (Gorbach *et al*. 2003*a*), with an appropriate threshold, IR imaging is able to assess complete attenuation in renal cortical blood flow, evoked by occlusion of the main renal artery and its increase during post-occlusion hyperaemia (figure 8). The fact that, during total renal occlusion, the IR technique demonstrated that the magnitude of the VSO temperature oscillations decreased more significantly than the magnitude of the TGF thermal oscillations might suggest that VSOs are more sensitive to ischaemia than TGF. More experiments with kidney occlusion may need to be conducted to investigate this phenomenon further.

The present study is the first to investigate VSOs of the rat kidney using IR imaging. The described imaging method can be applied to study the effects of temperature heterogeneity on both metabolic and vascular rhythmicity. Furthermore, by characterizing vasomotor tone in different parts of the vasculature, one can assess the local control of blood flow and the associated heterogeneity of endothelial function.

## 5. Conclusions

Two oscillation frequencies with different intensities can be observed using two methods (single-point tubular pressure and IR imaging) and are coherent for the VSO and TGF frequencies. The highest levels of TGF synchronization were confined to the immediate vicinities of the warmest areas of the kidney. Although the literature provides broad evidence for the existence of both metabolic and vascular-derived VSOs, at present we have no confirmed explanation for the source of the thermal oscillations, but we are inclined to believe their vascular nature. Through the visualization of the spatial extent of these oscillations, as well as their synchrony and propagation phenomena, IR imaging holds promise in assessing kidney autoregulatory mechanisms.
